# Global Ionospheric Modelling using Multi-GNSS: BeiDou, Galileo, GLONASS and GPS

**DOI:** 10.1038/srep33499

**Published:** 2016-09-15

**Authors:** Xiaodong Ren, Xiaohong Zhang, Weiliang Xie, Keke Zhang, Yongqiang Yuan, Xingxing Li

**Affiliations:** 1 School of Geodesy and Geomatics, Wuhan University, 129 Luoyu Road, 430079, Wuhan, Hubei, China; 2 Key Laboratory of Precise Engineering and Industry Surveying of National Administration of Surveying, Mapping and Geoinformation, 129 Luoyu Road, 430079, Wuhan, Hubei, China; 3 German Research Centre for Geosciences (GFZ), Telegrafenberg, 14473 Potsdam, Germany

## Abstract

The emergence of China’s Beidou, Europe’s Galileo and Russia’s GLONASS satellites has multiplied the number of ionospheric piercing points (IPP) offered by GPS alone. This provides great opportunities for deriving precise global ionospheric maps (GIMs) with high resolution to improve positioning accuracy and ionospheric monitoring capabilities. In this paper, the GIM is developed based on multi-GNSS (GPS, GLONASS, BeiDou and Galileo) observations in the current multi-constellation condition. The performance and contribution of multi-GNSS for ionospheric modelling are carefully analysed and evaluated. Multi-GNSS observations of over 300 stations from the Multi-GNSS Experiment (MGEX) and International GNSS Service (IGS) networks for two months are processed. The results show that the multi-GNSS GIM products are better than those of GIM products based on GPS-only. Differential code biases (DCB) are by-products of the multi-GNSS ionosphere modelling, the corresponding standard deviations (STDs) are 0.06 ns, 0.10 ns, 0.18 ns and 0.15 ns for GPS, GLONASS, BeiDou and Galileo, respectively in satellite, and the STDs for the receiver are approximately 0.2~0.4 ns. The single-frequency precise point positioning (SF-PPP) results indicate that the ionospheric modelling accuracy of the proposed method based on multi-GNSS observations is better than that of the current dual-system GIM in specific areas.

The ionosphere can be defined as the part of the upper atmosphere where the density of free electrons and ions is high enough to influence the propagation of electromagnetic radio frequency waves^1^. When signals travel from the satellite to the receiver, the ionosphere can change the signals’ speed and cause a delay in both the code and carrier phase observations. It is now believed that ionospheric delay is one of the main sources of error when positioning and navigating with GNSS^2^,^3^,^4^,^5^,^6^. To obtain a better position accuracy for positioning users, it is critical to have a precise ionospheric model. Related scientific studies of the ionosphere (e.g., ionospheric storms, ionospheric scintillation, and anomalous variations of geomagnetic storms, earthquakes and tsunamis) also require permanent and continuous monitoring of the ionospheric state^7^,^8^,^9^,^10^. Therefore, the question of how best to obtain a continuously precise ionospheric model with high spatial and temporal resolution on the global scale is an important issue for precise positioning and space weather applications.

The Global Positioning System (GPS), which can provide global coverage with simultaneity, time continuity, low operating expense and high temporal resolution for the users, has become an effective tool which is widely used to continuously monitor the Earth’s ionosphere with high spatial and temporal resolution^7^,^11^,^12^,^13^,^14^,^15^,^16^,^17^. To set up a global public service for monitoring ionospheric total electron content (TEC) based on ground GNSS receivers, the International GNSS Service (IGS) Working Group on Ionosphere was established in 1998^11^,^18^, which contains four IGS Ionospheric Associate Analysis Centres (IACCs): the Centre for Orbit Determination in Europe (CODE), Jet Propulsion Laboratory (JPL), European Space Agency (ESA), and Polytechnic University of Catalonia (UPC). They use different approaches to compute the global ionospheric model and the corresponding details of their techniques can be seen in these references^11^,^14^,^19^,^20^. In the past, the ionospheric modelling using GNSS was based on GPS observations alone^21^,^22^ or combined with GLONASS observations^18^,^23^. The GLONASS system (Russia), has been operating at full capability with 24 satellites since 2011. Like GPS, it can guarantee the user’s visibility of at least four satellites. Galileo, which is still under construction, is the European global satellite-based navigation system. The complete Galileo constellation will comprise 30 satellites spread evenly around three orbital planes inclined at an angle of 56 degrees to the equator (http://www.gsa.europa.eu/galileo/programme). BeiDou is a new system that China started constructing in 2000. It has already provided positioning, navigation, and timing services for the entire Asia Pacific region since 2012, and the ultimate aim of BeiDou is to have the capability of global coverage with its unique constellation consisting of five GEO (Geostationary Earth Orbit), three IGSO (Inclined Geo-Synchronous Orbit), and 27 MEO (Medium Earth Orbit) satellites in 2020 (http://www.beidou.gov.cn). At present, there are more than 80 satellites in orbit. When the four systems are all in full operation, there will be nearly 120 satellites available. Meanwhile, IGS has established a new MGEX network to collect and analyse data from GPS, GLONASS, BeiDou and Galileo. This network currently includes about 140 monitoring ground stations. Other GPS networks will be upgraded to multi-GNSS observation networks in a few years^24^,^25^; this means that more GNSS observations can be utilized, which will provide several times more IPP to effectively improve the spatial coverage of observations and the accuracy of ionospheric modelling.

Thanks to the development of BeiDou and Galileo, there are many recent studies of ionospheric modelling using GPS/BeiDou^26^,^27^, or GPS/Galileo combined observations^16^,^28^. In this contribution, we develop a global ionospheric model using multi-GNSS (GPS, GLONASS, BeiDou and Galileo) observations and assess its performance and accuracy. The processing and strategy we used to build the ionospheric model are presented in detail in Section 2. In Section 3, the accuracy and performance of the four-system model will be evaluated in many aspects: First, we perform a rigorous analysis about the distribution of IPP under different satellite system conditions. Then, to obtain a statistically representative evaluation and to explore the contribution of the multi-GNSS system, we compare our ionospheric model with the products derived from the IGS Analysis Centres (JPL, UPC, ESA and CODE). After that, we test the accuracy of SF-PPP using the estimated multi-GNSS GIM to assess the performance of the model further. Finally, we analyse the variation characteristics of DCB in satellite and receiver by comparing with that of other institutions, which can evaluate the accuracy of the ionospheric model from another angle.

## Method

### Slant Ionospheric measurements derived from Multi-GNSS

Because the ionosphere is a dispersive medium, the ionospheric refraction depends on the frequency of the signal. To be more specific, the ionospheric refraction is proportional to1/*f*^2^, where *f* is the carrier frequency, ignoring very small higher-order terms. For a dual-frequency GNSS receiver (eg.GPS, GLONASS, BeiDou and Galileo), taking the frequencies *f*_1_ and *f*_2_ as examples, the ionospheric delay measurement of the pseudorange code and carrier phase can be obtained as follows:


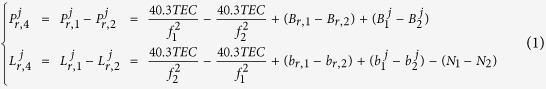


where *j* and *r* represent satellite and station, respectively; *P* and *L* are the pseudorange and carrier phase observations, respectively; TEC is the slant total electron content along the propagation path (in TECu); *f*_1_and *f*_2_ are the frequencies of the carrier phases of *L*_1_ and *L*_2_, respectively; *B*_*r*_and *B*^*j*^ stand for the receiver and satellite hardware delay of the pseudorange code (in m), respectively; *b*_*r*_ and *b*^*j*^ are defined as the receiver and satellite hardware delay of the carrier phase (in m), respectively; N is carrier-phase ambiguity (in m).

The ionospheric observations can be obtained directly by pseudorange code or carrier phase measurement. However, because of the pseudorange measurement noise, the precision of the carrier phase is much higher than that of the pseudorange, but there is an ambiguity parameter in carrier phase measurements, which would cause high complexity^19^. To reduce the noise of the pseudorange and to remove the ambiguity of the carrier phase, the so-called “levelling carrier to code” algorithms are used in this study.

The ionospheric observables of multi-GNSS (GPS, GLONASS, BeiDou and Galileo) can be obtained as follows:


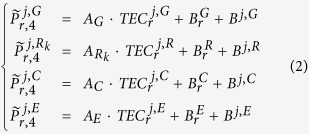


where 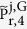, 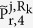, 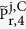 and 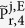 denote the code-levelled carrier phase ionospheric observables of GPS, GLONASS, BeiDou and Galileo, respectively. Because the frequencies are different for each satellite system, A_G_, A_C_ and A_E_ represent the constants that are used to convert TEC (in TECu) to length (in m) for GPS, BeiDou and Galileo, respectively. 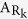 denotes the GLONASS satellite with frequency factor k that is used for the computation of the carrier phase frequencies of the individual GLONASS satellites; 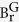, 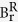, 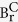 and 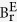 are the DCB of receiver for different satellite system, respectively; B^j,G^, B^j,R^, B^j,C^ and B^j,E^ represent the DCB of jth satellite for different satellite systems, respectively.

In equation (2), 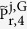, 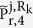, 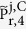 and 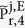 are observable values. A_G_, A_C_ and A_E_ represent the constants that are used to convert TEC (in TECu) to length (in m) for GPS, BeiDou and Galileo, respectively. TEC, 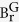, 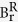, 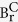, 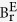, B^j,G^, B^j,R^, B^j,C^ and B^j,E^ represent unknowns.

When all of the satellites in the four GNSS systems are observed, equation (2) becomes singular. To separate the DCB of satellites and receivers, some additional external references must be introduced. A zero-mean condition for all satellites is adopted in this paper, as shown in equation (3).


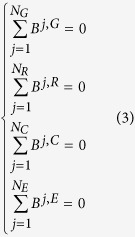


where *N*_*G*_, *N*_*R*_, *N*_*C*_ and *N*_*E*_ are the total number of observed satellites of the GPS, GLONASS, BeiDou and Galileo systems, respectively.

### Ionospheric modelling based on Multi-GNSS STEC measurements

To model the spatial distribution of TEC, the spherical harmonic expansion model based on the Single Layer Model (SLM) is used in this paper^15^:


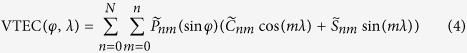


where φ and λ are the geomagnetic latitude and solar-fixed longitude of IPP, respectively; VTEC(φ, λ) is the vertical ionospheric TEC at the IPP VTEC(φ, λ), which is converted from slant TEC using a mapping function; N is the max degree of the spherical function (in this paper, N = 15 is used to model global ionospheric TEC; 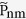 represents a regularization Legendre series of degree n and order m; 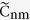 and 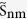 are spherical harmonic coefficients to be estimated.

From equations (2) and (4), a mapping function is needed to convert slant TEC to vertical TEC. The mapping function is expressed as follows:


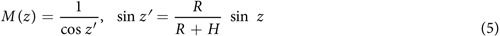


where *R* is the mean radius of the Earth, *H* = 450 *km* is the altitude of the single-layer ionosphere, z is satellite’s zenith distance at the corresponding receiver, *z*′ is satellite’s zenith distance at the corresponding IPP.

For the same satellite system, the weight of ionospheric observation at different elevations are calculated using the following formula:


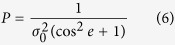


where e is the satellite elevation; *P* is the corresponding observation weight; 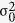 is the observation noise variance. The noises of the smoothed ionospheric measurement can be estimated as^14^,^29^,^30^


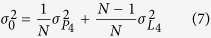


where N is the smoothing length. 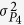
and 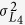
are the noises of TEC measurements derived from geometry-free combination of code and carrier phase observations, respectively, which are set as 3 TECu and 0.1 TECu.

For different satellite systems, we determined the weighting factors of GPS, GLONASS, Galileo and BeiDou based on the ionospheric observation accuracy of four systems in section 3.2.2. From the analysis results, the weighting factors of GPS, GLONASS, Galileo and BeiDou are 3, 1.5, 3 and 1, respectively.

In this study, we used an expansion of spherical harmonics up to the order of 15 with a 2-hour interval to model the global ionosphere maps in a solar-geomagnetic reference frame. A modified single-layer model mapping function was used to convert slant TEC into VTEC. The effective height has been selected to be 450 km. Piecewise linear functions were used for representation in the time domain. The DCB were estimated as constant values for each day, and the zero mean conditions for all satellites of each system were adopted to separate the DCB of satellites and receivers.

## Results and Analyses

### Data selection and processing strategy

**Figure 1:**
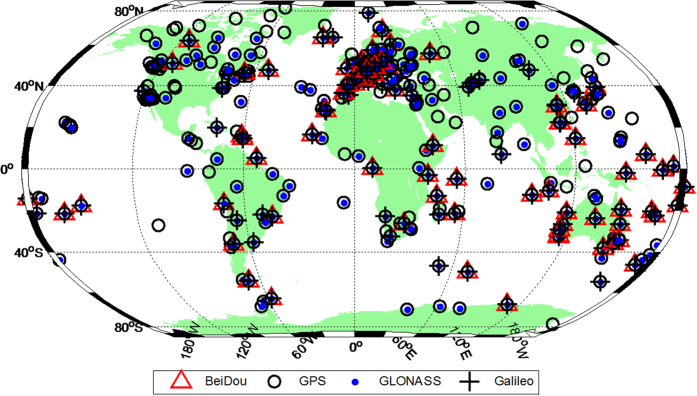
Distribution of reference stations tracking BeiDou (red triangle), GPS (black circle), GLONASS (blue dot) and Galileo (black cross). The figure is drawn by M_Map, which is a mapping package for Matlab (https://www.eoas.ubc.ca/~rich/map.html).

To evaluate the performance of global ionospheric modelling with multi-constellation (GPS, GLONASS, Galileo and BeiDou) observations, more than 300 stations (approximately 100 stations from the MGEX networks provided with GPS/GLONASS/Galileo/BeiDou observations and approximately 200 stations equipped with dual- or triple-frequency receivers from the IGS networks capable of tracking GPS or GLONASS satellites) were selected. The station distribution is shown in Fig. 1 drawn using Matlab2010b software. For ionospheric modelling, the observation types we used were P1 and P2 for GPS and GLONASS data, C1X and C5X for Galileo data, and C2I (corresponding to B1) and C7I (B2) for BDS data. Therefore, the computed DCB were P1–P2 for GPS and GLONASS, C1X-C5X for Galileo and B1–B2 for BDS. Sixty days (approximately two months) of Multi-GNSS observations from 21 May to 19 July 2015 (DOY 141 to 200) were recorded at a sampling rate of 30 seconds. To reduce multipath errors and the noise level of ionospheric measurements, the carrier-phase smoothed-code observations were used with an elevation mask of 10°.

### IPP distribution and accuracy of ionospheric observables

**Figure 2:**
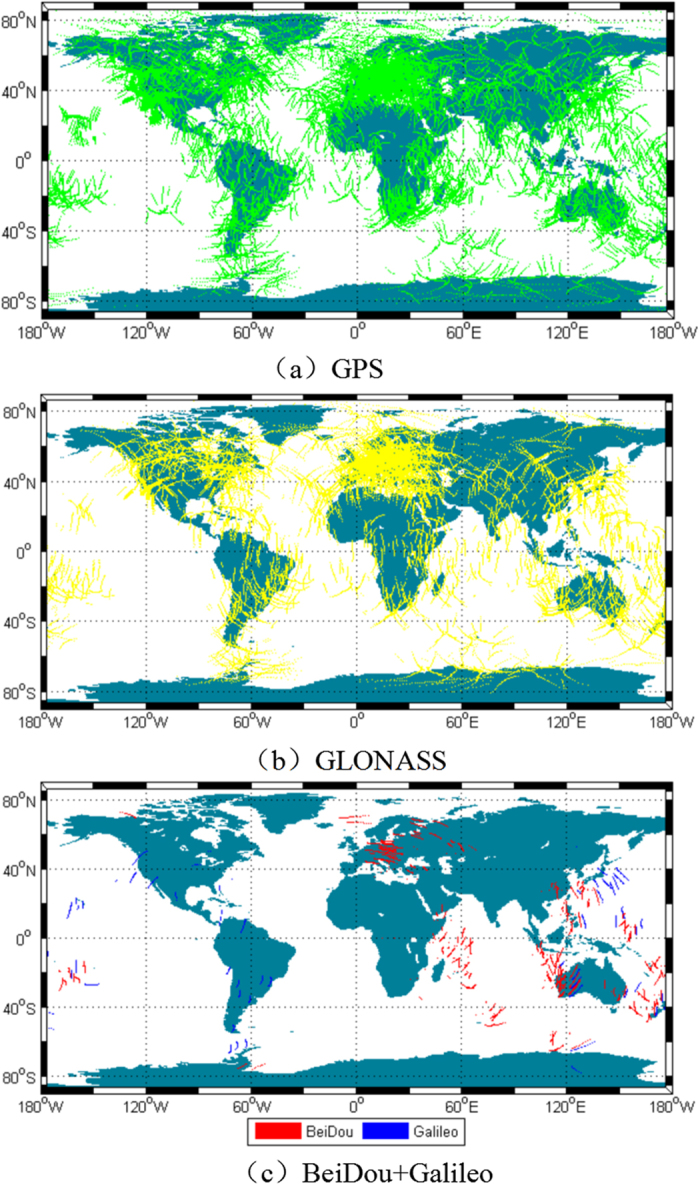
IPP distributions of GPS (red), GLONASS (blue), BeiDou (green) and Galileo (yellow) during 2 hours (UT00:00–02:00) on June 2, 2015. The figure is drawn by M_Map, which is a mapping package for Matlab (https://www.eoas.ubc.ca/~rich/map.html).

The characteristics of IPP and the accuracy of ionospheric observables for GPS, GLONASS, BeiDou and Galileo are analysed in this section. Figure 2 shows the IPP distribution of the four systems during 2 hours (UT00:00-02:00) on June 2, 2015. As seen in Fig. 2, the IPP distribution of GPS is apparently densest and mainly covers the majority of continental areas across the globe because of a large number of tracking stations and a full satellite constellation. With the completion of recovery for GLONASS, it has a good IPP distribution in most areas, and the number of IPPs is nearly as large as that of GPS. For the BeiDou system, its distribution is uneven, being mostly concentrated on Australia, East Asia and Europe. One of the main reasons for this finding is that there are currently only 13 operating satellites, and five of them are geostationary satellites, which cannot increase the spatial resolution of IPP distribution over time. In addition, the monitoring stations tracking BeiDou satellites are relatively limited and not evenly distributed, which causes the IPP number to be much less than that of GPS. Figure 2b shows that being limited by the number of satellites, the Galileo system has fewer IPPs globally than the BeiDou system. Undoubtedly, with increases in the number of Beidou and Galileo satellites and the upgrading of the IGS tracking network, there will be more and more MGEX stations and satellites of BeiDou and Galileo in orbit; this means that the IPP distribution will become very dense over most areas of the world. Furthermore, the capacity of ionospheric monitoring will be further improved as well.

The ionospheric observables are a sum of the true line-of-sight ionospheric slant total electron contents (STEC), DCB and other non-modelling errors, such as multipath. To clearly understand the magnitude of these errors, an experiment using the single-difference of ionospheric observables between two very close receivers (e.g., short- or zero- baselines) were performed by Ciraolo^31^. The true STEC between the two receivers and the same satellite is the same, and by performing the single-difference, a constant is theoretically derived as a result of the same receiver pairs DCB. As multipath error and code noise remain, different arcs may display different values, which can be an indicator of the accuracy and reliability of ionospheric observations.

**Figure 3:**
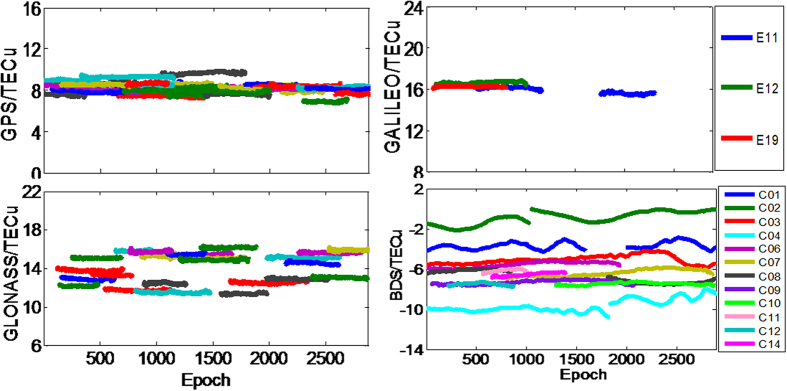
Single-difference (short baseline cut0-cut1) of the levelled carrier-phase ionospheric observables for different satellite systems (GPS/GLONASS/BeiDou/Galileo) at DOY 141, 2015.

To assess the accuracy and reliability of ionospheric observations from the four systems, we selected a set of data over a short-baseline CUT2-CUTC (DOY 141, 2015) at Curtin University in Australia. Figure 3 depicts the experiment result. The top-left panel shows that GPS satellites have the least peek-to-peek variation of approximately 3.2TECu, which leads to an extracting error of 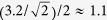 TECu. The 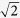 term is introduced by single-differencing of observations between two stations of short- or zero- baseline, and the denominator 2 indicates a 95% confidence level (2 sigma). Similarly, the extracting errors for GLONASS satellites (top-right) and BeiDou satellites (lower-right) are 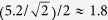 TECu and 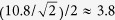 TECu, respectively. For Galileo satellites (upper-right), no evaluation can be made because of the limited number of satellites (the broadcast ephemeris of only three Galileo satellites was provided in that period).

### Ionospheric model accuracy

**Figure 4:**
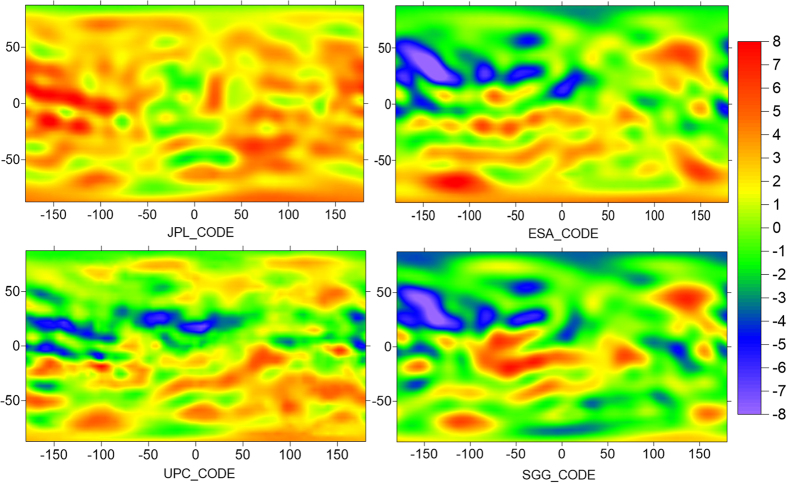
Grid map of VTEC biases of JPL, UPC, ESA and SGG with respect to CODE at epochs 00:00UT of DOY 151, 2015.

**Figure 5:**
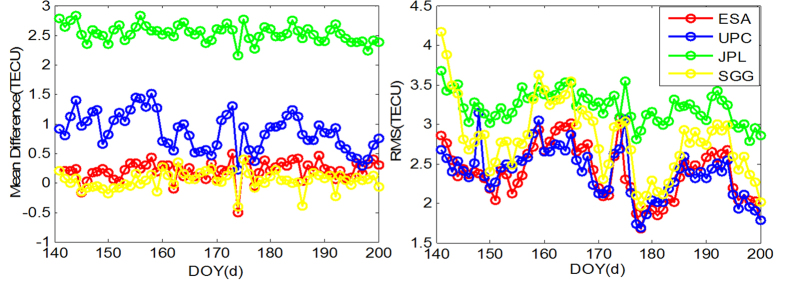
Mean difference (left) and RMS (right) values of GIM for JPL, UPC, ESA and SGG with respect to CODE from DOY 141 to DOY 200, 2015.

To further evaluate the estimated VTEC products, we processed multi-constellation ionospheric observations over sixty days (DOY 141 to 200 in 2015) to produce 5° * 2.5° global ionosphere maps at the School of Geodesy and Geomatics (SGG) of Wuhan University. This section mainly demonstrates the performance of SGG’s multi-GNSS GIM products and the model differences between SGG, JPL, UPC, ESA and CODE. The results are shown in Figs 4 and 5.

Figure 4 shows the VTEC differences of JPL, UPC, ESA and SGG compared with CODE at UT00:00 for DOY 151, 2015. It can be found that the VTEC products of these four institutions are in good agreement with CODE; the difference between them is less than 2 TECU over most of the area. The largest differences are mainly concentrated in the equatorial region with high ionospheric activities or in the ocean area where the stations are very sparse. It is shown that the ionospheric products of UPC, ESA and SGG are very close to CODE. However, the ionospheric products of JPL have a significant systematic bias compared with CODE, which is approximately 5 TECu. It is worth mentioning that the accuracy of CODE’s GIM is 2 to 8 TECu. Because the observations used to model the global VTEC by CODE are from GPS and GLONASS, the results of SGG show good consistency with CODE.

The statistical VTEC differences with respect to CODE during DOY 141 to 200, 2015 are presented in Fig. 5; it shows that the mean difference in VTEC maps between ESA and SGG is close to zero, while those values are approximately 1 TECu for UPC and 2.5 TECu for JPL. This is the systematic bias mentioned above and shown in Fig. 4. The RMS of GIM for ESA, UPC, JPL and SGG with respect to CODE are approximately 1.5–3 TECu, 1.5–3 TECu, 3–3.7 TECu and 2–4 TECu, respectively. The RMS of ESA, UPC and SGG are in good agreement with each other, except for a few days of SGG. The systematic bias of VTEC maps between JPL and CODE is nearly 3 TECu.

**Figure 6:**
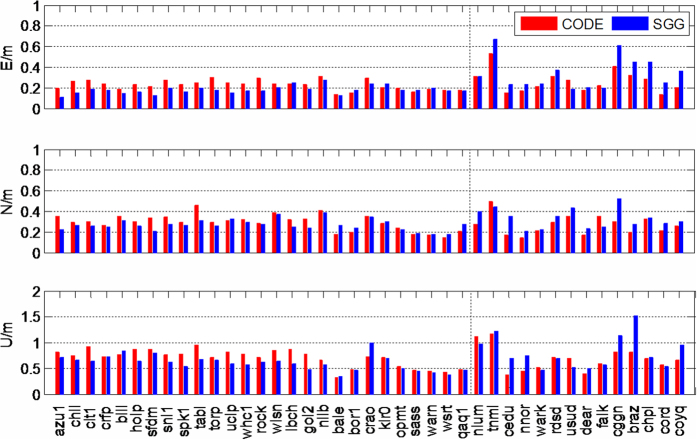
Positioning RMS of East (top), North (middle) and Up (bottom) components for 44 stations on DOY 141–200 2015, while using the ionosphere from CODE (red bar) and SGG (green bar).

**Figure 7:**
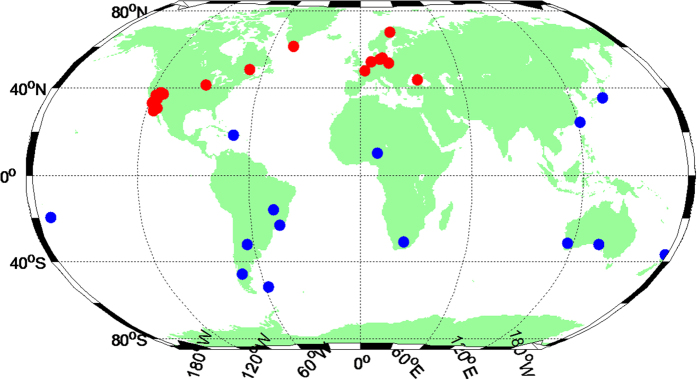
Distribution of testing stations. The figure is drawn by M_Map, which is a mapping package for Matlab (https://www.eoas.ubc.ca/~rich/map.html).

To further validate the estimated ionosphere products from multi-GNSS observations, we perform a kinematic single-frequency precise point positioning (SF-PPP) test using 41 global distributed stations. These stations are excluded in the GIM calculation. The average RMS of 41 stations for the north, east and up directions from DOY 141 to DOY 200 2015 are shown in Fig. 6. It shows that there is little difference across regions in positioning results. For a more simple explanation, we divided the selected stations into two parts by dotted line as shown in Fig. 6. The stations of the left side (the corresponding red dots in Fig. 7) show a relatively high accuracy while using SGG GIM, and there is no significant difference on the right side (the corresponding blue dots in Fig. 7) while using GIM products of CODE or SGG. The reason for these differences is that the left stations are mainly located in Europe and North America, which include the added IPPs of BeiDou and Galileo in these areas now. It is worth believing that the accuracy of SF-PPP will be significantly improved all over the world with the increase of BeiDou and Galileo IPPs in the future.

### Performance of multi-GNSS satellite and receiver DCB determination

DCB is the difference of the instrumental delays (the time delays between the antenna and the signal generating or signal processing units in the hardware of satellite and receiver) between two signals. Because DCB couples with the TEC, its accuracy and stability greatly affect the accuracy of estimated TEC. Therefore, DCB can be used for evaluating the accuracy of the ionospheric model. In this section, the variation characteristics of DCB in satellite and receiver for different GNSS systems are compared and assessed.

**Figure 8:**
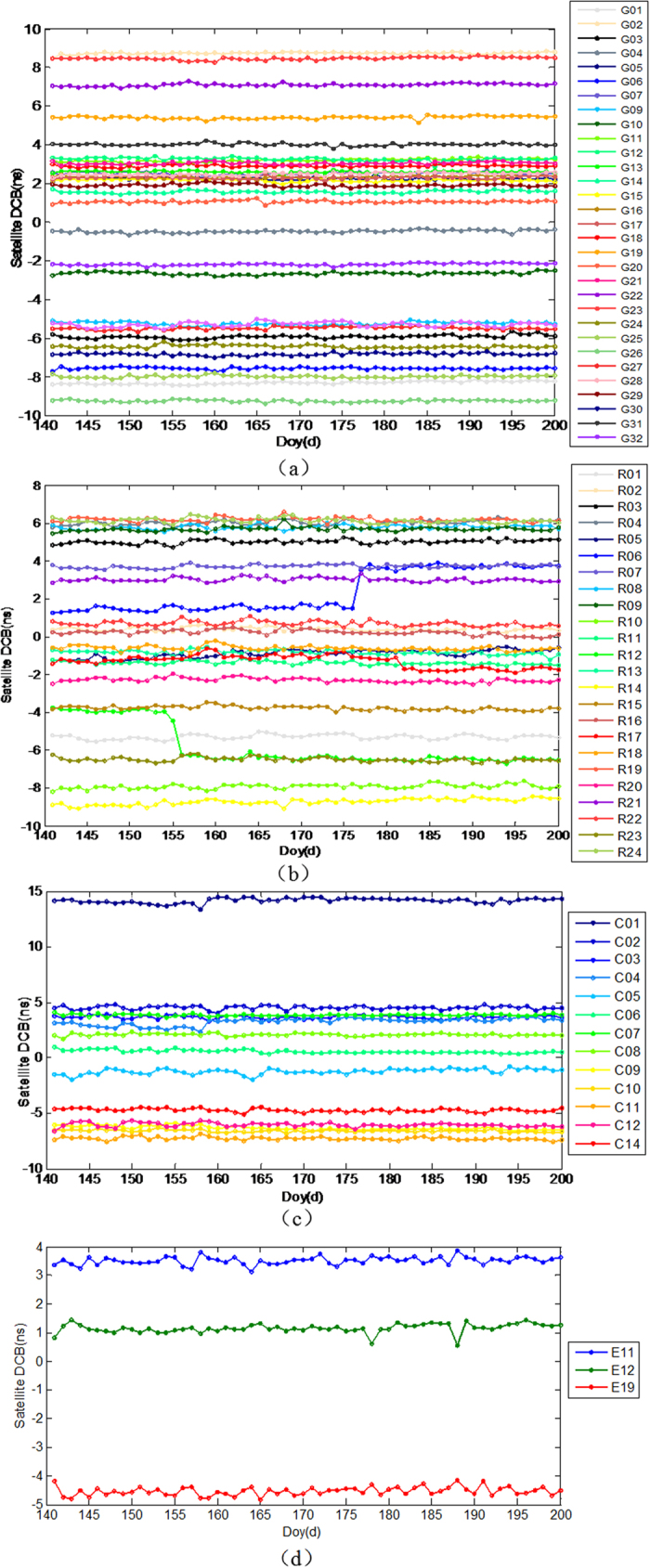
DCB time series for all satellites of different GNSS systems during DOY 141 to 200, 2015: (**a**) GPS DCB(P1-P2), (**b**) GLONASS DCB(P1-P2), (**c**) BeiDou DCB (C2I-C7I), (**d**) Galileo DCB(C1X-C5X).

The daily DCB estimates of GPS, GLONASS, Galileo and BeiDou during the period from DOY 140 to 200, 2015 are shown in Fig. 8a–d, respectively. It can be seen that the daily DCBs of all satellites appear to be fairly stable and the daily variation of all satellite’s DCBs are within 1 ns with the exceptions of R06 at DOY 176 and R12 at DOY 155. The jumps in the figures may be caused by hardware operations effects. The P1-P2 DCB values of GPS and GLONASS are in the range of −10 to 10 ns and −10 to 8 ns, respectively. The C2I-C7I DCB values of BeiDou are confined to a range of ±8 ns with the exception of GEO satellite C01 (approximately 15 ns), and the C1X-C5X DCB of Galileo are confined to a range of ±5 ns, which has good consistency with the results of Wang *et al.*^29^. It also can be seen that the GPS DCB is the most stable. To further evaluate the stability of the estimated DCB products, we compare the STD of estimated DCB with the daily DCB products from CODE and Institute of Geodesy and Geophysics (IGG)^29^,^30^.

**Figure 9:**
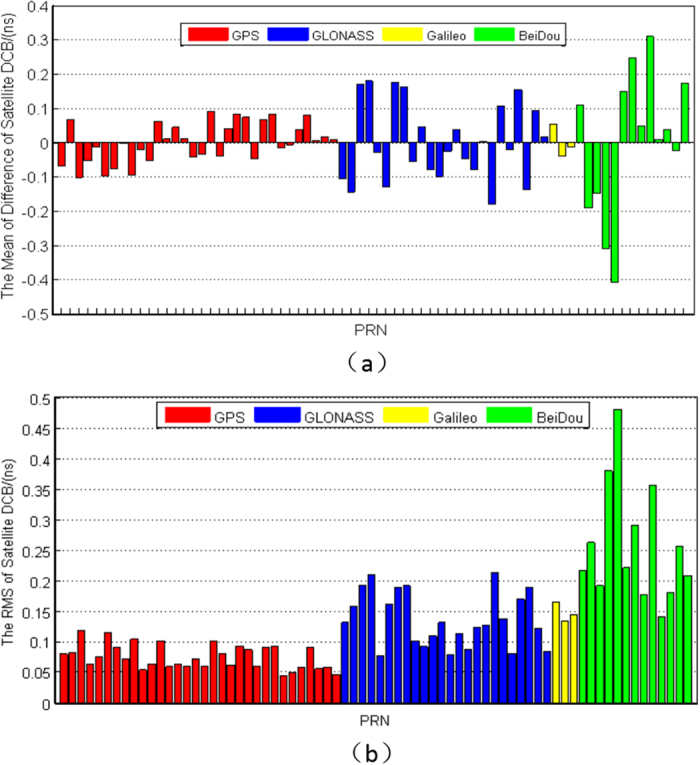
RMS of estimated satellite DCB compared with the daily DCB products of CODE/IGG from DOY 141 to 200, 2015: (**a**) mean offset; (**b**) root mean square error. The GPS, GLONASS, Galileo and BeiDou systems are shown by the red, blue, yellow and green bars, respectively.

The comparison of the estimated satellite DCB with CODE/IGG products during the study period are presented in Fig. 9. It can be seen from Fig. 9a that the mean offset of all GPS and Galileo satellites are within ±0.1 ns, those of GLONASS satellites are slightly higher than those of GPS and Galileo (within ±0.2 ns), and the BeiDou satellites have the largest mean offset, which is generally better than ±0.4 ns with respect to IGG. A similar phenomenon is displayed in Fig. 9b; it can be seen that the RMS of GPS and GLONASS satellites are generally within 0.1 ns and 0.2 ns, respectively. Compared with the mean offset, the RMS of Galileo is slightly poorer, but not more than 0.15 ns. Similarly, the RMS of BeiDou satellite is the largest; this may be due to the limited numbers of satellites and stations (see Fig. 8). It is obvious that the estimated satellite DCBs are reliable compared with the products of CODE/IGG.

**Figure 10:**
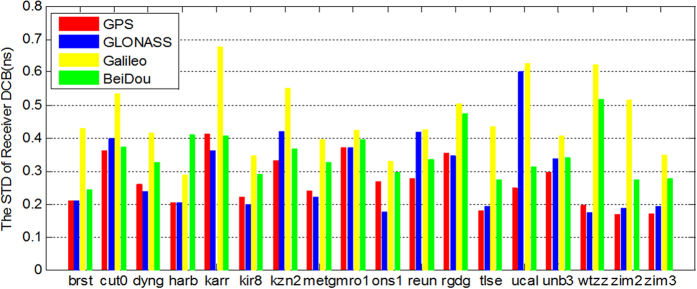
Standard deviations of multi-GNSS receiver DCB estimates at some MGEX stations for the entire time series (GPS: red; GLONASS: blue; Galileo: yellow and BeiDou: green).

The STD of receiver DCBs of 18 four-system MGEX stations are shown in Fig. 10. It can be seen that the STDs of receiver DCB are much larger than those of satellites, though their STD values are within 0.5 ns. The STD of the GPS receiver DCB is the lowest in four systems. Its value is approximately 0.2~0.3 ns for most of the stations. The STD of receiver DCB of GLONASS is slightly larger than that of GPS in general. Compared to the GPS and GLONASS receiver DCB, the BDS receiver DCB STD is larger than that of GPS and GLONASS. Galileo has the largest STD of the receiver DCB values, with values of up to 0.5 ns for most of the stations.

## Summary and Conclusions

In this paper, the global ionosphere map based on multi-GNSS observations was produced, and its modelling method was also described. By using 60 days of data (DOY 141 to 200, 2015) from the MGEX and IGS networks, we first analysed and compared the IPP distribution and accuracy of the ionospheric observables obtained from different systems. The accuracy and reliability of the estimated multi-GNSS ionosphere map and its by-product of DCB estimates were then assessed and analysed with respect to solutions from other institutions (CODE, JPL, UPC, ESA and IGG).

The results showed that the IPP distributions of GPS and GLONASS have a good global coverage. However, the IPP distributions with only observations from the BeiDou and Galileo systems, the distributions are uneven and mainly cover specific regions because of the limited numbers of satellites and stations. Despite all of this, the total number and spatial resolution of IPP are still improved to some extent. The accuracy of GPS ionospheric observables is the best while the accuracy with only BeiDou is much poorer than GPS and GLONASS. The DCB estimates of GPS, GLONASS, BeiDou and Galileo have good stability for both satellite and receiver DCBs, where the stability of GPS DCB is the best. The STD values for GPS, GLONASS, BeiDou and Galileo DCB are approximately 0.06 ns, 0.10 ns, 0.18 ns and 0.15 ns in satellite, respectively, and the corresponding STD values are all approximately 0.2~0.4 ns in the receiver, which is consistent with the DCB products of CODE/IGG. For the ionospheric model, the results show that our ionosphere products based on multi-GNSS observations are in good agreement with other institutions’ products based on GPS-only or GPS + GLONASS observations. The SF-PPP results indicate that the ionospheric modelling accuracy of the four-system is better than that based on single or dual systems. With the increased numbers of BeiDou and Galileo satellites and the upgrading of the IGS tracking networks, they are expected to further increase the capacity of ionosphere monitoring.

## Additional Information

**How to cite this article**: Ren, X. *et al.* Global Ionospheric Modelling using Multi-GNSS: BeiDou, Galileo, GLONASS and GPS. *Sci. Rep.* **6**, 33499; doi: 10.1038/srep33499 (2016).
